# Effects of CoQ10 on the erythrocyte and heart tissue cholinesterase, nitric oxide and malondialdehyde levels in acute organophosphate toxicity

**DOI:** 10.1186/cc9787

**Published:** 2011-03-11

**Authors:** A Bayır, H Kara, Ö Köylü, R Kocabaş, A Ak

**Affiliations:** 1 Selçuk University, Selçuklu Faculty of Medicine, Konya, Turkey; 2 State Hospital, Konya, Turkey; 3 Meram Trainin Hospital, Konya, Turkey; 4 Selçuk University, Meram Faculty of Medicine, Konya, Turkey

## Introduction

The aim of this study was to examine the effects of CoQ10 on malondialdehyde (MDA) and nitric oxide (NO) levels and on the choline esterase (CE) activity in the heart tissue and erythrocytes in acute organophosphate poisoning (AOP) and to compare it with antidote treatment.

## Methods

Twenty rabbits were divided into three groups as sham (*n *= 8), PAM-atropine (*n *= 6), and CoQ10 groups (*n *= 6). The blood samples were taken from each test subject to measure plasma and erythrocyte CE, NO, and MDA values before toxicity. To all of the groups were given 50 mg/kg DDVP by orogastric tube. After toxicity, venous blood samples were taken to establish post-toxicity plasma and erythrocyte CE, NO, and MDA levels in the first, 12th and 24th hours. The rabbits in the sham group did not receive treatment. The test subjects in the PAM-atropine group were given 0.05 mg/kg atropine with repeated doses when required and 30 mg/kg i.v. bolus, then 15 mg/kg PAM i.v. every 4 hours. The subjects in the CoQ10 group received 50 mg CoQ10 i.v. Thoracotomy was performed in the 24th hour on the subjects in all groups and heart tissue samples were obtained to evaluate CE, NO and MDA values in the tissues. The test subjects were given high-dose i.v. anesthesia and were sacrificed at the end of the study.

## Results

In the 12th and 24th hours erythrocyte CE levels of the CoQ10 group were considerably higher than the PAM-atropine group (*P *= 0.007, 0.017, respectively). It was established that erythrocyte MDA and NO levels of the CoQ10 group were significantly lower than the PAM-atropine group in the 12th and 24th hours (*P *< 0.05). Heart tissue CE levels of the CoQ10 group were considerably higher than the sham and PAM-atropine groups (*P *= 0.001). Heart tissue MDA and NO levels of the CoQ10 group were significantly lower than the sham and PAM-atropine groups (*P *= 0.000, 0.000, 0.001, 0.000, respectively).

## Conclusions

Treatment of AOP with CoQ10 plus PAM-atropine has a therapeutic effect on both erythrocyte and heart tissue lipid peroxidation and CE activity. Using CoQ10 with PAM-atropine in AOP patients with cardiac damage could reduce morbidity and mortality. Further clinical studies would be of benefit to clarify this matter.

